# A prospective analysis of robotic targeted MRI-US fusion prostate biopsy using the centroid targeting approach

**DOI:** 10.1007/s11701-019-00929-y

**Published:** 2019-02-19

**Authors:** Saiful Miah, Pol Servian, Amit Patel, Catherine Lovegrove, Lindsey Skelton, Taimur T. Shah, David Eldred-Evans, Manit Arya, Henry Tam, Hashim U. Ahmed, Mathias Winkler

**Affiliations:** 1 Department of Urology, Charing Cross Hospital, Imperial Healthcare NHS Trust, Fulham Palace Road, London, W6 8RF UK; 2 Division of Surgery and Interventional Science, University College London, 21 University Street, London, WC1E 6AU UK; 3 Department of Radiology, Charing Cross Hospital, Imperial Healthcare NHS Trust, Fulham Palace Road, London, W6 8RF UK; 4 Division of Surgery, Department of Surgery and Cancer, Faculty of Medicine, Imperial College London, London, SW7 2AZ UK

**Keywords:** Robotic prostate biopsy, Targeted biopsy, Fusion biopsy

## Abstract

Robotic prostate biopsy is an emerging technology. Recent development of this tool has allowed the performance of a transperineal prostate biopsy allowing pre-programmed standardized biopsy schemes. Prospective data collection was undertaken in 86 consecutive men who underwent robotically assisted transperineal prostate biopsy. All underwent a multi-parametric MRI pre-biopsy with centroid targeting followed by systematic template prostate biopsy. For the purposes of this study, our definition of clinically significant prostate cancer (csPCa) is any Gleason score > 6. Mean (SD) age, median (IQR) PSA, and median (IQR) prostate volume were 64.24 (6.97) years, of 7.79 ng/ml (6.5) and 45.06 cc (28), respectively. Overall, 44 (51.2%) men were diagnosed with csPCa. csPCa was detected in the targeted biopsies alone in 35 (40.1%) men. The addition of the 12-zone template biopsy increased the yield of csPCa for another 9 (10.5%) men. Of these 9 men, the majority (7) harbored primary pattern 3 disease and only 1 was identified to have high-grade disease. Out of these 9 men, 7 of them had the identification of csPCa in the sector, where a target was contained within that zone. Robotic-assisted prostate biopsy in our study has demonstrated a high detection of csPCa when combined with limited near-field sampling. Our study suggests the use of more accurate biopsy schemes such as ring-targeting of lesions to mitigate against systematic and random mathematical errors. Adoption of this tool and biopsy strategy would potentially avoid the increased morbidity associated with whole gland systematic unguided biopsies.

## Introduction

Prostate cancer remains a significant health burden and cause of male mortality. Detection of prostate cancer has evolved from blind systematic transrectal biopsies to MRI lesion directed transperineal biopsy including robotic approach such as employed here [1].

There is now level 1b evidence with the results of PROMIS demonstrating the clinical benefits of a pre-biopsy prostate MRI and using the transperineal (TP) route to procure prostate tissue for diagnostic purposes [2]. The TP route for prostate biopsy also allows the urologist to mitigate against the increasing prevalence of fluoroquinolone resistance attributed to the transrectal prostate biopsy [3]. The TP prostate biopsy does, however, have a steeper learning curve and when coupled with a pre-biopsy MRI requires potential additional soft- and hardware to allow MRI-US fusion. One such strategy to address and allow greater implementation of targeted TP prostate biopsy is the use of a robotic tool.

Robotic technology has revolutionized surgical treatment of prostate cancer with the therapeutic adoption of this tool globally [4]. Robotic prostate biopsy and needle localization are an emerging technology, which has shown potential to positively affect prostate cancer diagnosis and management [5]. However, current robotic technology in prostate cancer diagnostics almost exclusively uses the transrectal route for prostate biopsies [5].

The iSR’obot MonaLisa (Biobot Surgical Ltd, Singapore) is relatively unique in that it allows the performance of robotically assisted prostate biopsy with the added benefits of allowing it to be performed via the TP route.

Mathematical, systematic, and random errors have been measured in fusion biopsy systems and this should also be assumed to be present in percutaneous needle delivery devices used in robotic technology [6, 7]. Centroid targeting involves taking biopsies from the suspected tumor center. Ring targeting involves creating a ring around the suspected tumor where targets are spaced at equal arc lengths on the ring. Targeting strategies such as centroid versus ring targeting to mitigate against such errors on the robotic platform are yet to be addressed or investigated.

Here, we report on of the largest series of men who underwent an MRI-USS fusion robotically assisted TP prostate biopsy with centroid targeting. Our primary evaluation is the detection of clinically significant prostate cancer (csPCa) with robotic centroid targeting versus 12 sector systematic TP biopsy.

## Methods

### Patients

Prospective data collection was undertaken in 86 consecutive men who underwent robotically assisted TP prostate biopsy. Patient with a previous diagnosis of a prostate cancer was excluded from the study. All men were either a *de-novo* prostate biopsy undertaken for an elevated age adjusted PSA or abnormal DRE or had a previous negative transrectal ultrasound (TRUS) biopsy of their prostate elsewhere and were referred to our tertiary center for further evaluation and follow-up.

### mpMRI

All men underwent a multi-parametric MRI (mpMRI) carried out to minimal standards laid down by British Society of Uroradiology and the European Society of Uroradiology. That is, coronal or sagittal T2W pelvis, transverse T2W, multiple *b* value ADC, long *b* value (1500 or 2000 in 1.5 T or 3 T, respectively), and gadolinium enhanced dynamic contrast enhanced T1W axial scans. Images were reviewed by a board-certified radiologist (HT) and given a score using the PIRADS V2 scoring system 1–5. mpMRI images were uploaded into UroFusion (BioBot Surgical Ltd, Singapore) software which allowed delineation and contouring the prostate and suspected cancer lesions manually to generate a 3D model. Only men harboring PiRADs V2 score 3–5 lesions underwent a robotic-assisted prostate biopsy.

### Procedure—iSR’obot MonaLisa (Biobot)

**Fig. 1:**
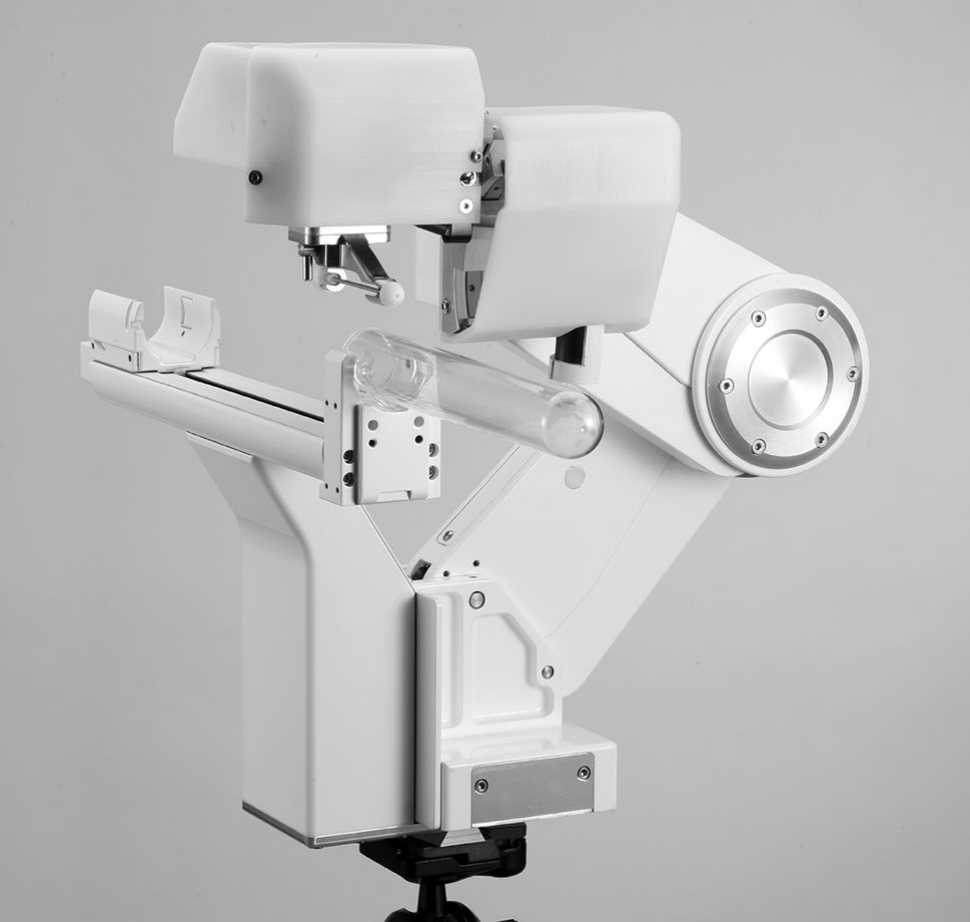
iSR’obot MonaLisa (Biobot Surgical Ltd, Singapore)

**Fig. 2:**
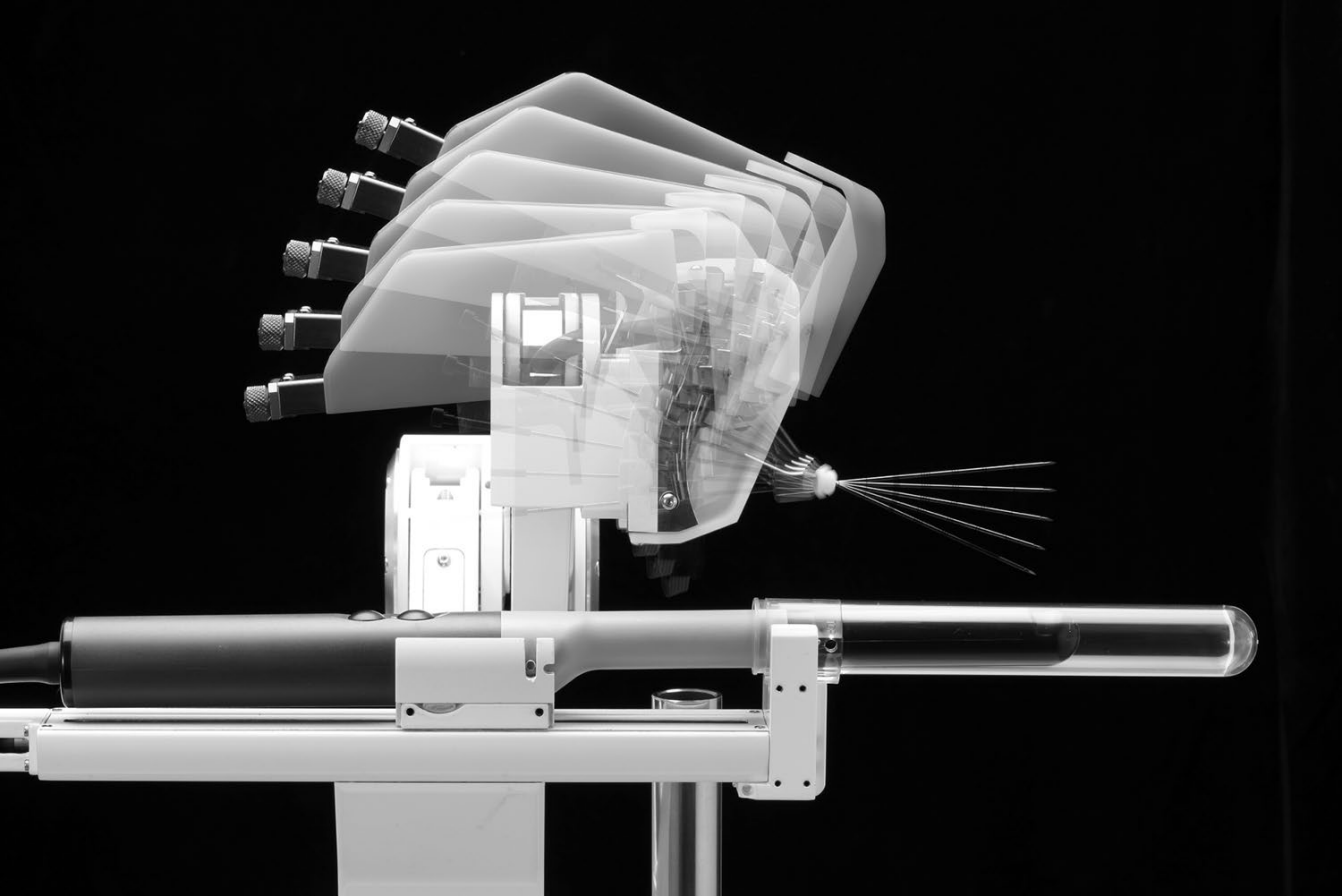
iSR’obot MonaLisa (Biobot Surgical Ltd, Singapore) with BK 3000 transrectal ultrasound probe

Biobot (Biobot surgical, Singapore) is an ultrasound-based robot for transperineal prostate biopsy (Figs. 1, 2). It incorporates a pre-biopsy MRI with real-time transrectal ultrasound images to construct a 3D model of the prostate. All MRI targets and prostate boundaries and were defined with UroFusion (HT) (BioBot Surgical Ltd, Singapore). The iSR’obot™ Mona Lisa robotic transperineal biopsy device utilizes a software-controlled robotic arm which we mounted onto the operation table via a Micro-Touch™ stabilizer. The MonaLisa system was connected to a BK3000 ultrasound machine with a transrectal probe which mounted onto the robotic arm to provide a TRUS image of the prostate. Uploading of the previously generated UroFusion 3-D prostate model and suspected cancer lesions was then performed. UroBiopsy (BioBot Surgical Ltd, Singapore) is then used to generate the TRUS-based model which allows fusion of the mpMRI, and TRUS models. The robot has a gantry that utilizes a double dual-cone concept to ensure that the entire prostate can be sampled with two 1 mm perineal skin punctures and also defines the penetration depth automatically. All procedures were done under general anaesthetic, and at induction, all patients received one dose of intravenous gentamicin 3–5 mg/kg according to local antibiotic guidelines. All patients underwent targeted biopsies first using centroid targeting of the lesion [7]. This was followed by a 12-zone template biopsy using a modified Barzell scheme.

### Outcomes

Our primary outcome was the detection of clinically significant prostate cancer (csPCa). Our secondary outcome included the rate of detection of csPCa for targeted and systematic sampling. For the purposes of this study, our definition of csPCa is any Gleason score > 6.

### Statistics

Descriptive statistics were used to describe the demographic data and prostate cancer detection rates. All data were entered into an Excel spreadsheet (Microsoft Excel Version 15.38) and an imported to STATA (version 13.1) for statistical analysis, in accordance with recommendations from the Standards of Reporting for MRI-targeted biopsy Studies (START) Guidelines [8].

### Ethics

Our study was reviewed and approved by our local Audit and Service evaluation board (Imperial College Healthcare, U.K: registration no. 298).

## Results

### Demographics

Mean (SD) age, median (IQR) PSA, and median (IQR) prostate volume were 64.24 (6.97) years, of 7.79 ng/ml (6.5) and 45.06 cc (28), respectively (Table 1). No man had more than 3 target lesions identified in the pre-biopsy mpMRI (range 1–3) with a median (IQR) number of targeted biopsy per patient of 8 (4). The median number (IQR) of non-targeted biopsy per patient was 20 (9).

**Table 1:** Patient demographics, radiological characteristics, and number of biopsies taken

	Mean	± SD	Median	IQR
Age (years)	64.24	6.97	63.51	10.63
PSA (ng/ml)	10.00	8.53	7.79	6.5
MRI prostate volume (cc)	51.03	25.24	45.06	28
MRI lesion volume (ml)	1.87	2.42	0.86	1.29
No. of lesions/patient (*n*)	1.40	0.56	1	1
No. of target biopsies/patient (*n*)	8.15	3.82	8	4
Target biopsy density (n/ml lesion vol)	9.28	10.36	5.15	10.59
Number of random unguided biopsies/patient	20.20	6.18	20	9

### Primary outcome

Overall, 44 (51.2%) men were diagnosed with csPCa (Table 2). In total, 116 target lesions were identified from our cohort of 86 patients. From the 116 MRI lesion identified, 49 (42.2%) were discovered to have csPCa (Table 3).

**Table 2:** Detection rate of cancer

	Significant cancer
Overall	44/86 (51.2%)
Target biopsy	35/86 (40.7%)
Exclusively present in random biopsy	9/86 (10.5%)

Significant cancer = Gleason score > 6

**Table 3:** MRI lesion-based detection rate of target biopsies

PiRADs V2 score	Significant cancer *n* (%)			Insignificant cancer *n* (%)
5	16/30 (53.3)	3 + 3	1	1/30 (3.3)
		3 + 4	9	
		4 + 3	5	
		8–10	2	
4	22/55 (40%)	3 + 3	5	5/55 (9.1)
		3 + 4	14	
		4 + 3	4	
		8–10	4	
3	0/22 (0)	3 + 3	2	2/22 (9.1)
		3 + 4	0	
		4 + 3	0	
		8–10	0	
Unclassified	2/9 (22.2)	3 + 3	0	0/9 (0)
		3 + 4	1	
		4 + 3	1	
		8–10	0	

Significant cancer = Gleason score > 6

### Secondary outcomes

csPCa was detected in the targeted biopsies alone in 35 (40.1%) men. The addition of the 12-zone template biopsy increased the yield of significant cancer for 9 (10.5%) men. Of these 9 men, the majority (7) harbored primary pattern 3 disease and only 1 was identified to have high-grade disease (Table 5). Out of these 9 men, 7 had the identification of csPCa in the sector, where a target was contained within that zone which suggests that the target was missed narrowly.

With respect to radiological scoring and pathological correlation, 16/30 (53.3%) PiRADs v2 score 5 lesions were discovered to harbor csPCa (Table 5). For PiRADs v2 scores 4 and 3 and 2, these figures were 22/55 (40%), 0/22 (0%), respectively.

The median maximum cancer core (MCCL) length for targeted biopsy was 8 mm versus 7 mm for cancer discovered in non-targeted biopsies which was non-significant.

There was one case of post-biopsy sepsis requiring hospital admission for intravenous antibiotic therapy which was the only major complication from this cohort of patients.

## Discussion

Our findings provide a timely evaluation of the utilization of robotic equipment in the prostate cancer diagnostic pathway that utilizes the clinically superior transperineal route. There has been an increased adoption of this technology in units globally but with minimal reported outcomes [9–11]. The detection range of csPCa for all the previous similar studies is 50.0%, 52.7%, and 61% which is in-keeping with our overall detection rate of 51.2%.

The results of PROMIS have now provided level 1 evidence for the application of pre-biopsy mpMRI [2]. This is rapidly becoming the standard of care throughout major institutions across the U.K and beyond with other centers closely following suite due to a national strategy to deliver this change [12]. It has been proposed that the introduction of a robotic tool would potentially shorten the learning curve when faced with the challenges of mpMRI-US fusion prostate biopsies especially when being performed outside the centers of excellence [11].

With respect to the clinical utility of the performance of unguided systematic over targeted biopsies, our data demonstrate that an additional 9 men (10.5%) had the exclusive presence of csPCa in these non-targeted cores. The median maximum MCCL was 6 mm for this cohort which was lower than that for targeted biopsies (median MCCL = 8 mm) (Tables 4, 5). The treatment modality for this cohort of patients was radical prostatectomy [*n* = 3 (3.5%)], external beam radical radiotherapy [*n* = 3 (3.5%)], and active surveillance (*n* = 3 (3.5%)).

**Table 4:** Gleason scores of target prostate biopsies

Disease–Gleason classification	Target biopsy *n* (%)	Histological characteristics	
3 + 3	8/116 (6.9)	MCCL med	4.5
		MCCL IQR	5.25
3 + 4	24/116 (20.7)	MCCL med	7.5
		MCCL IQR	6
4 + 3	19/116 (16.4)	MCCL med	9.5
		MCCL IQR	8
8–10	6/116 (5.2)	MCCL med	8
		MCCL IQR	5

*MCCL* = maximum cancer core length in mm

**Table 5:** Demographics and pathology of the nine patients discovered to have clinically significant disease cancer (Gleason score > 6) exclusively in non-target samples

	Range	Med	Mean
Age	55.6–73.1	63	63.7
PSA	3.4–24	7.3	8.9
Pros size (cc)	25–68	44	44.8
MCCL	1–8	6	5.3

Gleason score	*n*/9
3 + 4	7/9
4 + 3	1/9
4 + 4	1/9

*MCCL* maximum cancer core length in mm

Our clinical utility of unguided systematic over targeted biopsies suggests a significant miss-rate; however, this is lower than the 17% reported by Mischinger et al. [11]. However, the majority of the men in our study had the presence of this csPCa within the zone, where the target was contained suggesting only a near-field miss. Within our cohort of men, if a targeted biopsy in addition to limited random sampling of only the zones, where the targets where present would have resulted in only 2 men (2.3%) being missed for the presence of csPCa, a more acceptable error rate for this diagnostic test. The performance of template prostate biopsies where a large number of cores are taken in a systematic non-targeted manner has been shown to result in a high urinary retention rate and a detrimental impact on genitourinary functional outcomes, including deterioration in urinary flow and sexual function [13]. The employment of this robotic tool where biopsies are undertaken in a targeted manner with random samples only in the zone within or around the target would avoid excessive unguided prostate sampling. Spacing a minimum of three targeted samples around a lesion results in a considerably improved yield in simulation experiments compared to a centroid approach as utilized in this study [7]. The ring-targeting approach accounts and mitigates for guidance system, image registration and random errors that all accompany MRI-US fusion prostate biopsies [7].

Limiting the number of cores taken will result in efficiency- and cost-savings with the reduction of the number of consumables used with subsequent decrease in the burden of technical time and workload for our pathology departments [14, 15]. The future application and development of such a robotic tool could potentially combine biopsy and focal ablation into one session when using an accurate real-time tissue characterization modality.

This robotic tool does have disadvantages namely time- and cost-related factors. The workflow of robotically assisted transperineal prostate biopsy is significantly longer due the nature of needing to delineate the targets prior to biopsy and then longer real-time workflow of the machine in comparison with a free-hand transperineal biopsy. There is no provision of a sagittal view of the needle trajectory. The procedure does not lend itself easily to the local anaesthetic approach of a transperineal prostate biopsy due to the longer intervention time that is associated with a general anaestheic procedure [12]. However, a recent update of the iSR’obot MonaLisa (Biobot Surgical Ltd, Singapore) had incorporated motion compensation within its software which may potentially mitigate against this issue.

Our study limitations include the heterogeneous nature of our cohort of men which included those with the previous negative prostate biopsy and men who underwent this diagnostic test for the first time. Our study has a small size; however, with the exception of study by Mischinger et al. [11], our data report on a larger series than found in the literature [9, 10]. All men underwent the procedure under general anesthesia. This is not in-keeping with global practice, where the majority of prostate biopsies are done under local anesthesia within an office setting.

In conclusion, the robotic-assisted prostate biopsy in our study has demonstrated a high detection of csPCa when combined with only limited near-field sampling. Our study suggests that the use of ring-targeting of prostate lesions may mitigate against systematic and random mathematical errors associated with MRI-US fusion prostate biopsies. Adoption of this tool and biopsy strategy would potentially avoid the increased morbidity associated with whole gland systematic unguided biopsies. Furthermore, it may allow users who are relatively new to mpMRI-USS fusion prostate biopsies to perform this with added technological security whilst on their learning curve for this procedure.
